# Lung Ventilation: Technegas

**DOI:** 10.2967/jnmt.125.269769

**Published:** 2025-06

**Authors:** Tina M. Buehner

**TO THE EDITOR:** I am writing in response to the article titled “Ventilation Lung Imaging: Technegas” by Farrell et al. (1). Although the article offers valuable insights, I would like to address inaccuracies that may misinform readers.

**Under Rationale:** The article states that “Technegas (Cyclomedica Asia Pacific) is a system for producing ^99m^Tc-based ventilation studies using a carbon-based nanoparticle.” In fact, Technegas is not a system itself; rather, it is a radioaerosolized particle manufactured within a system called the Technegas Plus System. Additionally, the correct subsidiary in the United States is Cyclomedica USA, not Asia Pacific.

It is also mentioned that “Technegas does not produce aerosolized particles,” whereas Technegas is indeed a submicron radioaerosolized particle (2).

**Under Clinical Indications:** The indications that you have specifically listed may be department-specific and acceptable for off-label use within those department; however, the U.S. Food and Drug Administration (FDA) label (September 2023) approves Technegas (2) as follows:

TECHNEGAS, when used with sodium pertechnetate Tc 99m in the Technegas Plus System, provides technetium Tc 99m-labeled carbon inhalation aerosol (Technegas Aerosol), for use in adults and pediatric patients aged 6 y and older for:

- visualization of pulmonary ventilation
- evaluation of pulmonary embolism when paired with perfusion imaging

The indication of pulmonary ventilation can very well encompass some of the other indications you have listed but should be explicitly stated as such. Please see the package insert for full prescribing information (2).

**Under Contraindications:** The authors mention that “Pregnancy must be excluded according to local institutional policy. If the patient is breastfeeding, appropriate radiation safety instructions should be provided” and “Recent nuclear medicine study (radiopharmaceutical-dependent),” both statements of which are inaccurate. Studies have shown that Technegas V/P SPECT imaging can be used in pregnant patients and is often preferred because of its lower radiation dose to breast tissue (3). Per the U.S. FDA label, there are no contraindications associated with Technegas Aerosol (2). Misinformation here could delay care for pregnant women at increased risk for pulmonary embolism.

**Under Patient Preparation/Education:** The authors state that “The practice session should be done under the same conditions as the actual ventilation procedure, including position (upright or supine) and using the nose clip.” Per the U.S. FDA–approved package insert, it is recommended “To facilitate uniform delivery of the aerosol from the apex-to-base of the lungs, perform the administration with the patient in the supine position” (2).

**Under System Preparation:** “Connect and turn on the argon supply flow rate to 15 L/min.” The correct amount of argon is 18 L/min as per the onsite training program.

**Under Crucible Preparation:** The authors state “Add 200–900 MBq of ^99m^Tc-pertechnetate in 0.13–0.17 mL with the well vertical, but do not overfill the crucible.” Per the U.S. FDA–approved package insert, the recommended activity to be added to the crucible is 400–1,000 MBq (10.8–27 mCi) to achieve a lung count rate between 1,500 and 2,500 counts per second at the end of the last respiration. Additionally, the recommended volume of sodium pertechnetate required to produce Technegas is 0.1 mL not to exceed a maximum volume of 0.13 mL (2).

**Under Simmer:** Please note that, under U.S. FDA approval, it is referred to as a Technegas Plus System and not a generator in the United States.

**Under Patient Ventilation:** The authors state that “When a rate of 1,500–2,500 counts/s in the posterior position is achieved, release the delivery knob and allow the patient to take 1–3 breaths through the tube to clear the residual.” Per the U.S. FDA label “When the adequate pulmonary counts are achieved for imaging, the patient must continue exhaling air through the filter equipped exhalation circuit of the PAS for 5 breaths to 6 breaths to trap residual aerosol being exhaled” (2).

**Under Adjunct Imaging/Interventions:** The authors state that “Bronchodilator therapy can improve study accuracy in patients with acute obstructive lung disease,” which was applicable to other radioaerosolized particles used in the United States that were much larger in size and often displayed extensive central airway deposition of tracer. The small size and hydrophobic nature of Technegas particles is one of the advantages of using it compared with other tracers and therefore does not require interventions of adjunctive medications during imaging.

I commend the authors for promoting knowledge of Technegas for technologists and nuclear medicine departments; however, its unique characteristics must be understood to ensure accurate protocols and optimal imaging quality.

Thank you for considering these corrections. I trust that the *JNMT* is committed to maintaining high standards of accuracy and integrity.

## DISCLOSURE

Tina Buehner is employed by Cyclomedica USA as the Director of Clinical Affairs. No other potential conflict of interest relevant to this article was reported.

## Notes

**Tina M. Buehner***

**Cyclomedica USA, Atlanta, Georgia*

*University of Arizona, Tucson, Arizona E-mail: tmbuehner@arizona.edu*
